# Sex Differences in Oxidative Stress and Depression-Like Behavior Following Chronic Lead Exposure from Pregnancy to Adulthood

**DOI:** 10.1007/s12011-026-05283-3

**Published:** 2026-08-15

**Authors:** Nancy Sánchez-Fuentes, Francisca Pérez-Severiano, Leticia Carrizales Yáñez, Carolina López-Rubalcava

**Affiliations:** 1 Departamento de Farmacobiología, Centro de Investigación y de Estudios, Avanzados del IPN, Unidad Sede Sur, Calzada de los Tenorios No. 235, Granjas Coapa, Tlalpan, 14330 CDMX, México; 2 Laboratorio de Neurofarmacología Molecular y Nanotecnología, Instituto Nacional de Neurología y Neurocirugía Manuel Velasco Suárez, Insurgentes Sur No. 3877, La Fama. Tlalpan, 14269 CDMX, México; 3 Facultad de Medicina, Universidad Autónoma de San Luis Potosí, Av. Venustiano Carranza 2405, Lomas los Filtros, 78210 San Luis Potosí, SLP, México

**Keywords:** Lead exposure, Forced swimming test, Tail suspension test, Oxidative stress, Depression-Like behavior

## Abstract

Lead (Pb) is a neurotoxic heavy metal that crosses the placental barrier and may affect fetal development. However, the effects of chronic developmental Pb exposure on oxidative stress, stress-related endocrine responses, and depression-like behavior, particularly regarding sex differences, remain unclear. This study evaluated the effects of chronic Pb exposure from gestation to adulthood on redox balance, corticosterone responses, and depression-like behavior in male and female mice. Pregnant C57BL/6J mice received 250 ppm lead acetate in drinking water from gestational day 0 until offspring reached adulthood. Depression-like behavior was assessed using the forced swimming and tail suspension tests. Reactive oxygen species and lipid peroxidation were measured in the prefrontal cortex and hippocampus, and plasma corticosterone was quantified following behavioral stress. Chronic Pb exposure increased immobility in both behavioral tests and increased oxidative stress markers in the prefrontal cortex and hippocampus, with sex- and region-dependent differences. Pb exposure also altered corticosterone dynamics in both sexes: males showed a delayed corticosterone peak, whereas females exhibited a prolonged response that remained elevated 24 h after stress. Overall, chronic developmental Pb exposure was associated with depression-like behavior, oxidative imbalance, and altered corticosterone responses in a sex-dependent manner. These findings highlight biological sex as an important factor influencing the neurobehavioral, redox, and stress-related endocrine consequences of chronic developmental Pb exposure.

## Introduction

Lead (Pb) is a toxic metal commonly found in the environment due to industrial emissions from factories and foundries, mining activities, and natural deposits. It can be found in household items such as paint, cosmetics, plumbing materials, batteries, glazed ceramics, and enamel-coated clay [1, 2]. The primary routes of exposure to Pb are inhalation and ingestion [3]. Once absorbed, Pb binds to erythrocytes, displacing iron. It is then distributed via the bloodstream to the liver, kidneys, brain, and other organs. Pb can cross both the blood-brain barrier [4] and the placental barrier. This can result in the accumulation of Pb in the fetus [2].

Subacute and chronic Pb exposure can result in anemia, hypertension, renal dysfunction, and other signs of organ damage. It has also been linked to the progression of neurodegenerative diseases such as Alzheimer’s, Parkinson’s, and schizophrenia [5]. Additionally, Pb exposure has been linked to attention deficit hyperactivity disorder (ADHD) [6] and affective disorders such as anxiety [7, 8] and depression [9, 10]. Several studies have reported a significant association between elevated blood Pb levels and depressive symptoms, particularly among individuals with occupational exposure [11, 12].

Oxidative stress (OS) is considered one of the major mechanisms involved in Pb neurotoxicity. Pb exposure decreases levels of endogenous antioxidants, such as glutathione (GSH), while increasing levels of reactive oxygen species (ROS), leading to neuronal damage and dysfunction [13]. Beyond direct oxidative damage, growing evidence suggests that redox imbalance interacts with neuroendocrine and neurotransmitter systems involved in mood regulation, thereby providing a mechanistic link between environmental toxicants and affective disorders.

Sex differences play a significant role in susceptibility to and severity of Pb exposure. Children may experience more severe consequences from lead exposure than adults, and both experimental models and clinical studies have demonstrated sex dependent variability in behavioral and molecular outcomes [14]. In particular, sex-specific differences in inflammatory signaling, epigenetic regulation, and hormonal modulation of stress responses have been proposed as key determinants of vulnerability to Pb neurotoxicity [15, 16]. Experimental studies have shown that developmental Pb exposure induces differential behavioral and molecular alterations in males and females, including changes in gene expression and neuroinflammatory pathways [14, 15].

Most of the evidence linking Pb exposure to sex-specific depression-like behavior comes from animal models. For example [17], showed that exposure to Pb during development causes depression-like behavior in female rats. Additionally, Pb-induced neurotoxicity has been associated with molecular and genetic changes that vary by sex in experimental models. Epigenetic alterations caused by Pb exposure have been reported to be sex-dependent [18]. Overall, chronic exposure to Pb has been associated with the development of depression and anxiety-like behaviors in rodent models [19]. One study found that exposing male rats to 500 ppm of Pb caused signs of depression and impaired object recognition memory [19]. These depressive behaviors in rodents have been linked to mechanisms such as oxidative stress and alterations in neurotransmitter systems, including the serotonergic and nitrergic pathways [20, 21].

Major depressive disorder is currently understood as a multifactorial condition involving dysregulation of interconnected systems, including monoaminergic neurotransmission, the hypothalamic-pituitary-adrenal (HPA) axis, neuroinflammatory processes, and oxidative stress pathways. While the monoaminergic hypothesis has guided antidepressant development, more recent evidence indicates that alterations in stress-axis reactivity and redox balance play a central role in the pathophysiology of depression. The HPA axis is a major neuroendocrine system involved in the physiological response to stress, with corticosterone representing the principal glucocorticoid released in rodents following HPA-axis activation. Previous experimental evidence indicates that Pb exposure can alter corticosterone responses, suggesting that disruption of neuroendocrine stress regulation may represent an additional component of Pb-induced neurotoxicity. Thus, evaluating the temporal corticosterone response to a behavioral stressor may provide insight into whether chronic developmental Pb exposure alters stress-related neuroendocrine responses [22, 23]. Clinical studies have shown that women report higher levels of depression and anxiety symptoms than men, suggesting that sex dependent neurobiological responses to stressors may influence vulnerability to affective disorders [24].

Given this evidence, it is important to determine whether Pb exposure induces depression-like behavior and to clarify the mechanisms involved. In particular, an integrative approach that examines oxidative stress alongside hypothalamic-pituitary-adrenal (HPA) axis function may provide valuable insights into how environmental toxicants contribute to affective dysfunction.

Therefore, there is a need for experimental models that evaluate behavioral changes resulting from chronic Pb exposure in adults and distinguish altered behavior in males and females.

The aim of the present study was to evaluate whether chronic Pb exposure from gestation to adulthood alters redox homeostasis and stress-related neuroendocrine responses associated with depression-like behavior in mice. The study also aimed to highlight sex-dependent neurotoxic susceptibility. We specifically sought to determine whether these effects exhibit sex-dependent patterns across key limbic regions, including the prefrontal cortex and hippocampus. We hypothesized that chronic developmental Pb exposure would induce sex-dependent oxidative damage and dysregulation of corticosterone responses, thereby contributing to depression-like behaviors.

## Materials and Methods

### Drugs and Chemicals

Lead acetate was purchased from Fermont (Monterrey, México); nitric acid and hydrogen peroxide aqueous solutions were provided by J.T. Baker (Radnor, Pennsylvania, USA); both methanol and chloroform were also sourced from J.T. Baker. Quinine was acquired from Sigma-Aldrich (Toluca, Mexico), and 2’,7’-dichlorodihydrofluorescein diacetate (H2DCFDA) was purchased from Thermo Fisher Scientific (Waltham, Massachusetts, USA).

### Animals and Experimental Design

This study included 108 male and 108 female C57/BL mice. The animals were provided by the Department of Pharmacobiology at CINVESTAV-Sede Sur. The mice were housed in a temperature-controlled room with an inverted 12:12 light-dark cycle and had free access to water and standard food (LabDiet 5008, manufactured by PMI Nutrition International, LLC).

The study protocol (ethics code #0356 − 23) was approved by the Committee for the Care and Use of Laboratory Animals at CINVESTAV and was conducted in accordance with the National Institutes of Health Guide for the Care and Use of Laboratory Animals (NIH Publications) and the Mexican Official Standard for the Management and Care of Animals (NOM-062-ZOO-1999).

**Fig. 1:**
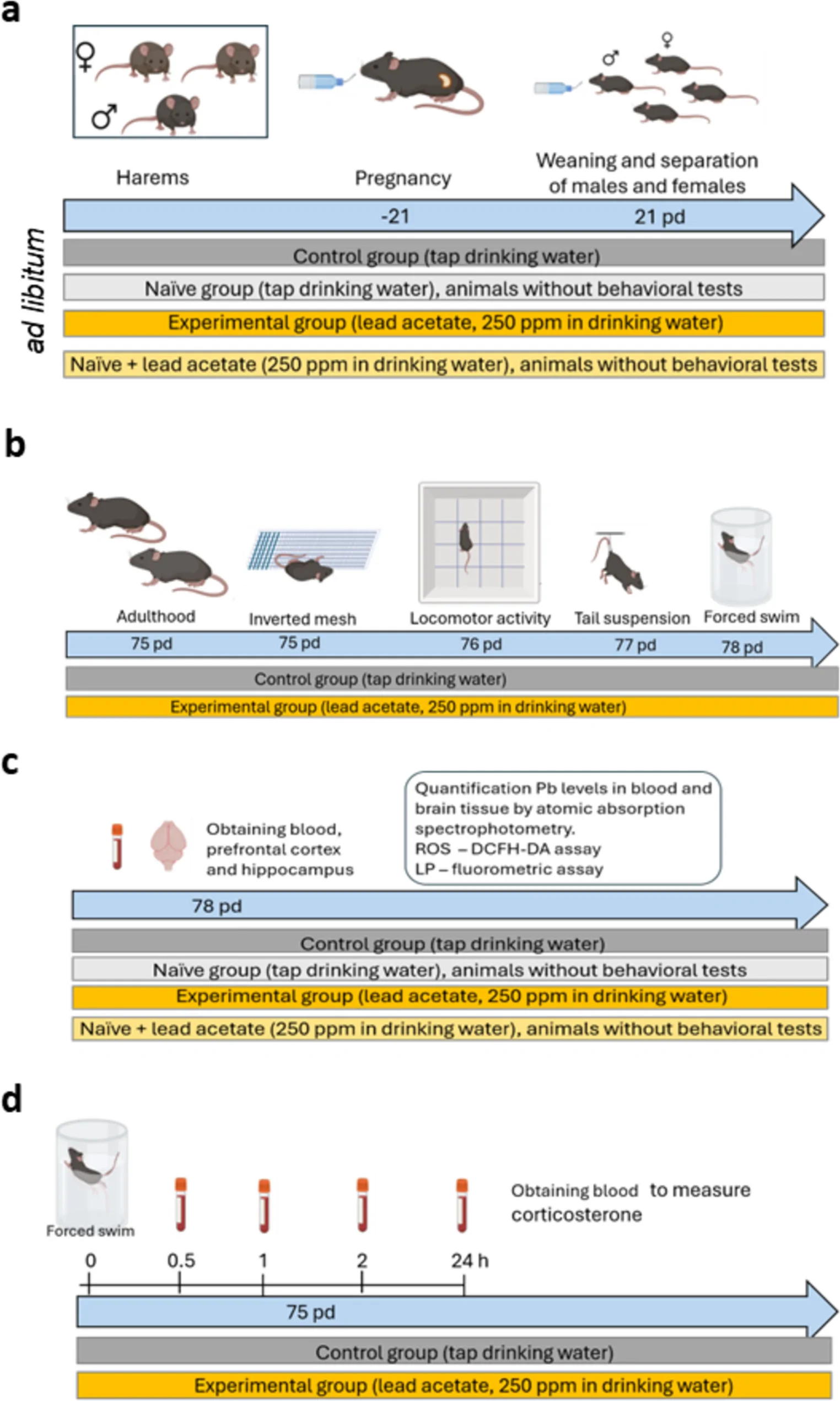
Experimental design, illustrating the formation of groups exposed to lead, the behavioral assessments performed, and the assays performed on brain tissue and blood. (**a**) Harem formation and Pb exposure; (**b**) Assessment of behavioral functions in adult mice and depression tests; (**c**) Dissection of brain tissue (prefrontal cortex and hippocampus); (**d**) Corticosterone curve. Abbreviations: dp, postnatal day; ROS, reactive oxygen species; DCFH-DA, 2′,7′-dichlorofluorescin diacetate; LP, lipid peroxidation. Image made by Biorender

Figure 1 illustrates the experimental design used in this work. The study began with the formation of harems consisting of two females and one male. From gestational day 0 until weaning, pregnant dams were exposed to either 250 ppm of lead acetate in their drinking water or were provided with tap water *ad libitum*. The Pb concentration was chosen based on previous experimental models showing that exposure to 250 ppm of Pb results in brain accumulation without overt systemic toxicity [25, 26].

After weaning, one offspring from each litter and sex was randomly assigned to each experimental group to prevent litter-related bias, resulting in the formation of four groups: (1) naïve (no behavioral tests + tap water); (2) naïve (no behavioral tests) + lead acetate 250 ppm; (3) control (behavioral tests + tap water); (4) Pb (behavioral tests + lead acetate) (Fig. 1a). The pups continued to receive either tap water or acetate lead until the end of the experiment. Behavioral function tests (inverted mesh and locomotor activity) were performed on day 75 postnatal (pd). To evaluate depression-like behavior, the animals underwent tail suspension (TST) and forced swimming tests (FST, Fig. 1b). Immediately after behavioral testing, the mice were sacrificed by decapitation to collect blood and brain tissue. The groups that did not undergo behavioral testing (naïve, naïve + lead acetate) were sacrificed by decapitation at 78 pd. Blood and brain tissue samples (from the prefrontal cortex and hippocampus) were obtained and used to quantify Pb levels through atomic absorption spectrophotometry. Reactive oxygen species (ROS) formation and lipid peroxidation (LP) levels in the prefrontal cortex and hippocampus were also evaluated (Fig. 1c). A separate group of mice was used to plot the corticosterone curve. The animals were tested in the FST at 75 pd and subsequently decapitated at different times (0, 0.5, 1, 2 and 24 h) to extract blood (Fig. 1d). The researchers performing behavioral scoring and biochemical assays were blinded to group assignment.

### Pb Quantification in Blood and Brain Tissue

Blood was collected immediately after decapitation. Then, it was treated with a diammonium hydrogen phosphate/Triton X-100 solution as a matrix modifier in the presence of 0.2% nitric acid (Suprapur^®^, Merck, Darmstadt, Germany). All samples were assayed using an atomic absorption spectrophotometer PinAAcle 900T (PerkinElmer MA, USA) with a graphite furnace following the Subramain method. The reference standard ClinChek¹ Level III Whole Blood Control (Labortechnick 212) was used for quality control. Each sample was assayed in duplicate. The recovery rate for the reference standard was over 95% [27].

Brain tissue samples from the prefrontal cortex and hippocampus were digested in a mixture of 50% nitric acid and 1 mL of hydrogen peroxide for 20 min in a MARS 6 microwave 200 °C/208 atm/20 min (CEM, Matthews, NC, USA) using atomic absorption-grade nitric acid and hydrogen peroxide. The Gelatin Multicomponent Trace Element reference standard (KODAK Laboratory, NY, USA) was used for quality control. Each sample was assayed in duplicate. The recovery rate for the reference standard was over 92%. Glassware and other materials were soaked in 10% nitric acid, rinsed with double-distilled water, and dried before use.

### Assessment of Behavioral Functions

#### Inverted Mesh Test

The Inverted Mesh Test was used to evaluate muscle strength. The test was performed when the mouse was 75 pd. A rectangular mesh measuring 36 by 26 centimeters with 0.5-centimeter squares was placed 50 centimeters high. The mouse was placed in the center of the mesh and rotated upside down. The latency time before the mouse fell was then measured [28].

#### Locomotor Activity Test

The Locomotor Activity Test is used to rule out alterations in general activity that could affect performance on behavioral tests. The apparatus is an acrylic box measuring 53 × 43 × 20 cm. The floor is divided into 12 equal squares. At 76 pd, the mouse was placed in a corner of the box for 10 min. The number of squares crossed with all four legs was counted [29].

#### Tail Suspension Test

The Tail Suspension Test (TST) is a behavioral assessment used to screen potential antidepressant drugs and evaluate interventions that may influence depression-like behaviors [30]. In this test, mice at 77 pd are suspended by their tails with adhesive tape, holding them in a straight position for 5 min. The duration of immobility is recorded. An increase in immobility time indicates increased depression-like behavior [31].

#### Forced Swimming Test

The Forced Swimming Test (FST) is a well-known behavioral paradigm used to evaluate the antidepressant-like effects of substances in rodent models. In this test, mice are placed individually in a glass cylinder (height: 24 cm; diameter: 13 cm) filled with 10 cm of water at 25 °C. The mice were allowed to swim for 6 min. Their behavior was videotaped during this time and scored later by an observer who was unaware of the experimental manipulations. After the test, each mouse was removed from the cylinder, dried with paper towels, and placed in a heated cage. Thirty minutes later, the mice were returned to their home cages.

The evaluated parameters were immobility behavior, during which the mouse makes no attempt to escape and only moves to keep its head above water; swimming behavior, which involves horizontal movement throughout the cylinder; and climbing behavior, during which the mouse hits the cylinder wall with its front paws. Since immobility is an indicator of depression-like behavior, an increase in immobility was interpreted as an increase in depression-like behavior [32, 33].

### Quantification of Reactive Oxygen Species

The production of reactive oxygen species (ROS) was determined using the 2’,7’-dichlorodihydrofluorescein diacetate (H2DCFDA) method. The brain areas were homogenized in physiological saline. Then, a TRIS-HEPES buffer solution (18:1) and 50 µL of 50 µM H2DCFDA were added. The samples were placed in a Synergy HT microplate reader (BioTek Instruments) at 37 °C for 60 min, with an excitation wavelength of 488 nm and an emission wavelength of 525 nm. A DCF calibration curve was used for ROS quantification. The results are expressed as pmol DCF/mg of protein/60 min [34].

### Lipid Peroxidation Measurement

The brain areas were homogenized in physiological saline. Then, a 900-µL sample aliquot was added to 4 mL of a chloroform/methanol mixture (2:1). Thirty minutes later, the organic phase was analyzed using a PerkinElmer LS55B luminescent spectrophotometer at excitation and emission wavelengths of 370 and 430 nm, respectively. Sensitivity was adjusted with quinine (0.001 mg/mL). Fluorescence values were corrected for the protein content of each sample [35]. Results are expressed as fluorescence units (FU)/mg of protein.

### Corticosterone Time-Course Curve

A separate group of mice was used to plot the corticosterone curve, with five mice tested at each time point. The animals were tested in the FST at 75 pd and subsequently decapitated at different times to extract blood. Serum corticosterone levels were quantified using an ELISA kit (Enzo, ADI-900-097, NY, USA).

### Statistical Analysis

Data are reported as the mean ± standard error (SEM). The groups were assessed using a two-way analysis of variance (ANOVA), followed by a post hoc Tukey test. Post hoc tests using the Dunnett method were applied to the corticosterone curve. A Pearson correlation test was performed to evaluate the relationship between immobility in the FST and ROS and LP levels. For behavioral tests, *n* = 12, and for biochemical tests, *n* = 5–6. Statistical significance was defined as *p* ≤ 0.05. No observations were excluded as outliers, and all data obtained were included in the statistical analyses. All statistical analyses were performed using either SigmaPlot 12.0 or GraphPad Prism 8.

## Results

### Pb in Blood and Brain Tissue

**Fig. 2:**
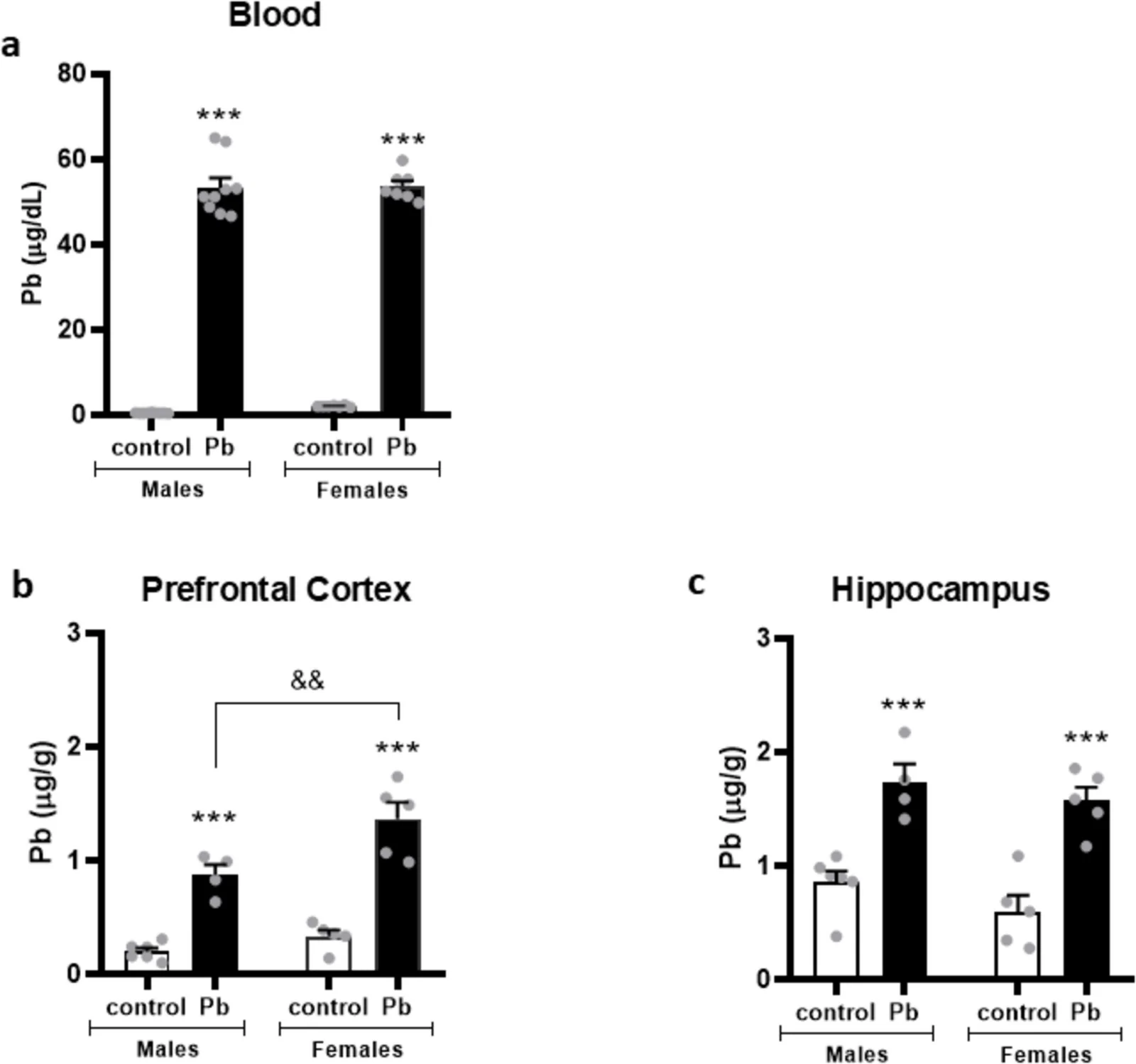
Quantification of Pb levels (*n* = 7–9) in blood (**a**), prefrontal cortex (5–6) (**b**), and hippocampus (5–6) (**c**). From the prenatal stage to adulthood, mice were exposed to either tap water (control) or Pb acetate (250 ppm) in their drinking water. The mice were sacrificed after behavioral testing (78 pd). Data are reported as the mean ± SEM and were analyzed using a two-way ANOVA followed by a post hoc Tukey test. *** *p <* 0.001 vs. control; $$$ *p <* 0.001 vs. males

Blood Pb levels were significantly higher in both female and male mice exposed to Pb (*F*(1, 28) = 1401, *p* < 0.001; Fig. 2a). Lead levels in the prefrontal cortex and hippocampus were also significantly increased (*F*(1, 16) = 94.4, *p* < 0.001 and *F*(1, 16) = 50.8, *p* < 0.001, respectively). These results indicate that the mice exposed to Pb had higher levels of this metal than the control group. Interestingly, female mice had higher Pb levels in their prefrontal cortex than male mice did (*p* = 0.007; Fig. 2b).

### Assessment of Behavioral Functions

#### Inverted Mesh and Locomotory Test

The Inverted Mesh Test, which evaluates muscle strength, revealed no significant differences between the experimental groups (*F*(1, 44) = 0.188, *p* = 0.17). Similarly, no significant differences were observed in the open field test, which evaluated locomotor activity (*F*(1, 44) = 2.065, *p* = 0.15). Together, these results suggest that Pb exposure may not significantly impact motor skills in mice (Table 1).

**Table 1:** Effect of chronic Pb on behavioral functions on the inverted mesh and locomotor activity tests in adult mice at 75 and 76 postnatal days.

Inverted mesh		
Latency of falling (s)	Control	Pb
Males	175 ± 3.3	162.9 ± 9.8
Females	170 ± 6.0	163 ± 8.0
Locomotor Activity		
Number of crossings	Control	Pb
Males	144 ± 9.5	142.7 ± 8.0
Females	130.2 ± 8.2	156.4 ± 7.4

The data are presented as mean ± SEM, n=12

### Depression Assessment

**Fig. 3:**
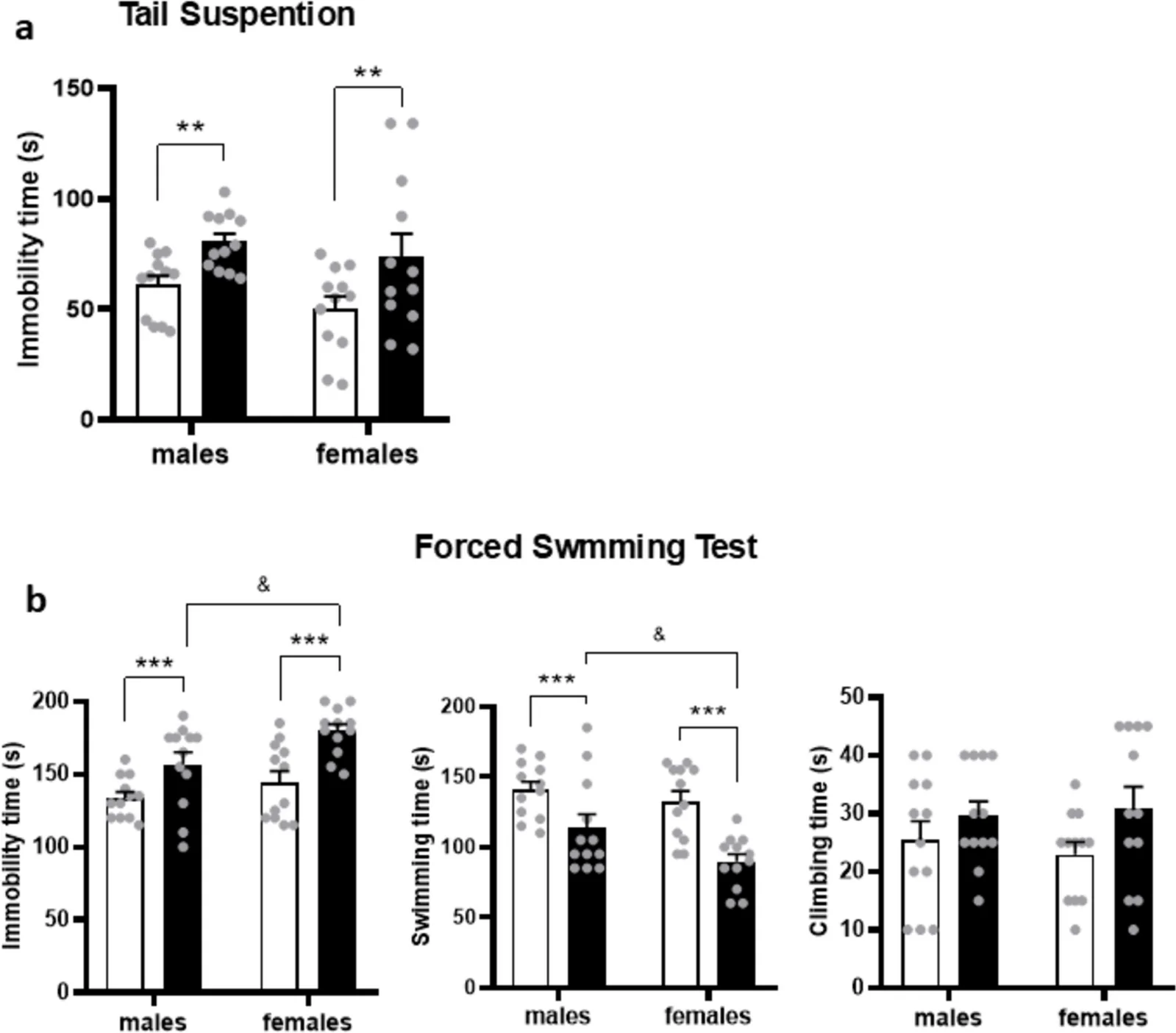
Evaluation of the tail suspension and forced swimming tests in male and female mice at 77 and 78 pd. The mice were exposed to either tap water (control, white bars) or Pb acetate (250 ppm, black bars) in their drinking water from the prenatal stage through adulthood. Data are reported as mean ± SEM (*n* = 12) and were analyzed using a two-way ANOVA followed by a post hoc Tukey test. *<*p* = 0.05; ***<*p* = 0.001 vs. control group; &*< p* = 0.05 vs. males

The Pb-exposed group exhibited greater immobility time in the TST than the control group (*F*(1, 44) = 11.2, *p* = 0.002), with significant differences observed for both males and females (*p* ≤ 0.001; Fig. 3a). In the FST, all Pb-exposed groups showed longer immobility times than the control group (*F*(1, 44) = 20.2, *p* = 0.001). Additionally, the Pb-exposed groups exhibited decreased swimming time (*F*(1, 44) = 23.1, *p* ≤ 0.001) in both males and females (Fig. 3b). Regarding climbing behavior, no differences were found between the experimental groups (*F*(1, 44) = 3.5, *p* = 0.065). Overall, adult male and female mice exposed to Pb exhibited increased depression-like behavior in both tests. However, the effects of Pb on female mice in the FST were significantly greater than those on males.

### Oxidative Damage: ROS and LP Levels

#### ROS in Brain Regions

**Fig. 4:**
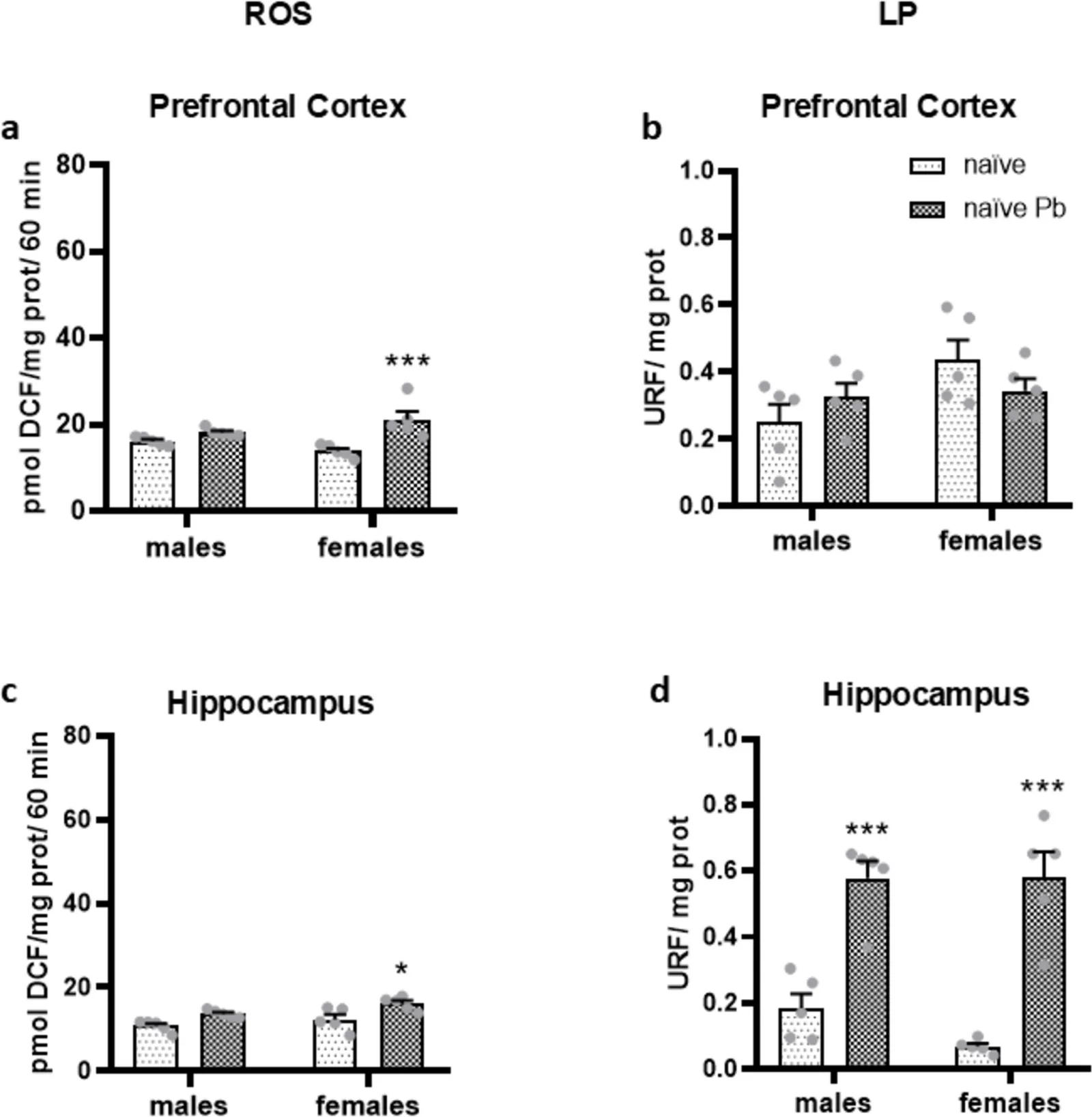
Oxidative damage to the brain tissue of mice that were not subjected to behavioral tests. Male and female adult mice were exposed to either tap water (control) or 250 ppm of Pb in their drinking water on postnatal day 75. The levels of reactive oxygen species (ROS) in the prefrontal cortex (**a**) and the hippocampus (**c**). The lipoperoxidation (LP) in the prefrontal cortex (**b**) and the hippocampus (**d**). Data are reported as mean ± SEM (*n* = 5–6) and were analyzed using a two-way ANOVA followed by a post hoc Tukey test. *<*p* = 0.05; **<*p* = 0.01; ***<*p* = 0.001 vs. control group

Once the animals reached 75 pd, brain tissue from specific regions was collected to assess oxidative damage by quantifying ROS and LP. Among animals that did not undergo behavioral testing (i.e., naïve animals), only females exposed to Pb exhibited significantly higher levels of ROS in the prefrontal cortex (*p* < 0.001) and the hippocampus (*p* < 0.012) (Fig. 4a and c).

**Fig. 5:**
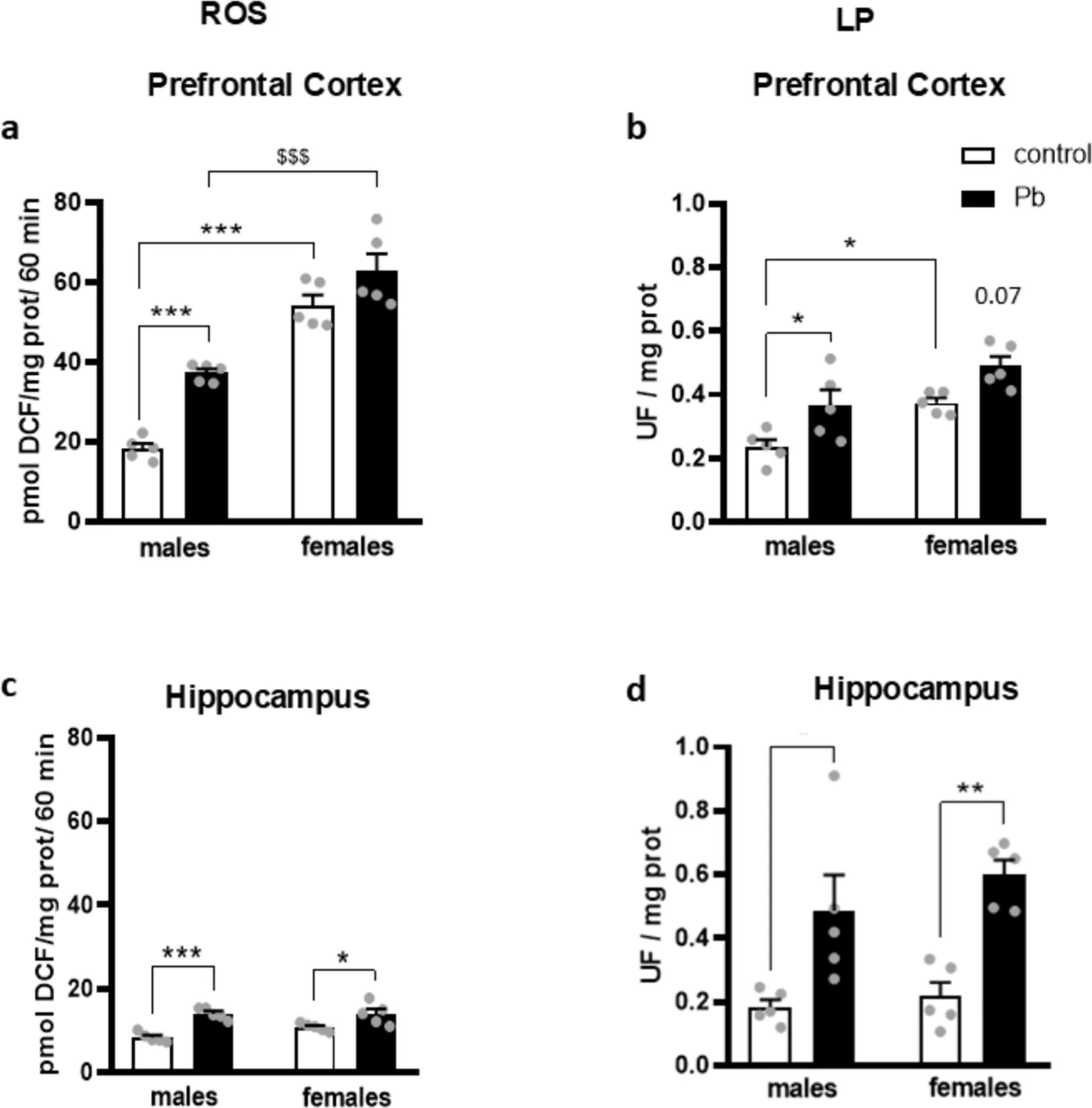
Oxidative damage to the brain tissue of mice subjected to the forced swimming test. Male and female adult mice were exposed to either tap water (control) or 250 ppm of Pb in their drinking water at 75 pd. The levels of reactive oxygen species (ROS) in the prefrontal cortex (**a**) and the hippocampus (**c**). The lipoperoxidation (LP) in the prefrontal cortex (**b**) and the hippocampus (**d**). Data are reported as mean ± SEM (*n* = 5–6) and were analyzed using a two-way ANOVA followed by a post hoc Tukey test. *<*p* = 0.05, **<*p* = 0.01 ***<*p* = 0.001 vs. control group; $$$<*p* = 0.001 vs. males

Meanwhile, male and female mice exposed to Pb and subjected to behavioral tests exhibited higher ROS levels in the prefrontal cortex than the control group (*p* < 0.001), and interestingly, in this cerebral region, females exposed to Pb exhibited significantly higher ROS levels than males (*p* < 0.001; Fig. 5a).

In the hippocampus, the groups exposed to Pb showed increased ROS levels compared to the control group (*F*(1, 16) = 36.44, *p* < 0.001). This increase was observed analogous in both male (*p* < 0.001) and female (*p* = 0.033) subjects (Fig. 5c).

#### LP in Brain Regions

No significant changes in LP levels were observed in the prefrontal cortex of naïve mice, not exposed to behavioral tests (*F*(1, 16) = 0.025, *p* = 0.87; Fig. 4b). However, the hippocampus of naïve animals exposed to Pb showed significantly higher LP levels than the control group (*F*(1, 16) = 76, *p* < 0.001; Fig. 4d).

The female control group in behavioral tests exhibited higher levels of LP in the prefrontal cortex than their male counterparts (*p* = 0.029; Fig. 5b). Interestingly, males exposed to Pb had significantly higher LP levels than the control group (*p* = 0.039). Additionally, females exposed to Pb exhibited a tendency toward increased LP levels, though it was not statistically significant (*p* = 0.07; Fig. 5b).

Exposure to Pb increased LP levels in the hippocampus of both male and female mice compared to the control group (Fig. 5d). This increase was statistically significant for both males (*p* < 0.001) and females (*p* = 0.003; Fig. 5d).

### Corticosterone Time-Course Curve

**Fig. 6:**
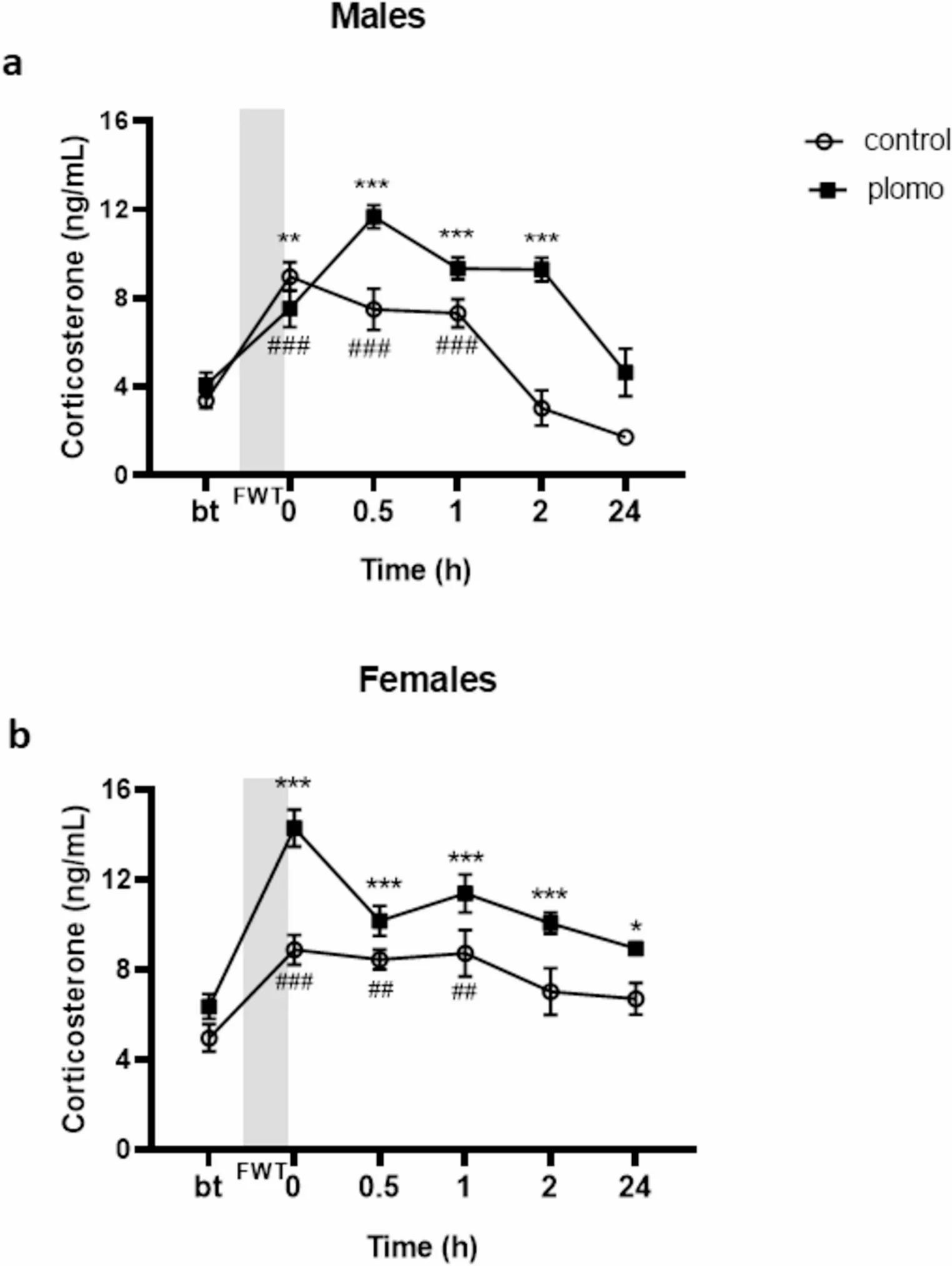
Time course of corticosterone serum levels. Male mice (Panel **a**) and female mice (Panel **b**) were exposed to either tap water (control) or 250 ppm of Pb in their drinking water at 75 pd. On day 75, the forced swimming test (FST) was performed. Then, blood samples were collected before the test (bt) and at different times to quantify serum corticosterone. Data are reported as mean ± SEM (*n* = 5–6 from each time point) and were analyzed using a two-way ANOVA followed by a post hoc Dunnet test. *<*p* = 0.05; ***<*p* = 0.001 vs. bt-Pb; ##<*p* = 0.01, ###<*p* = 0.001 vs. bt control group

Corticosterone serum levels were measured in a separate group of animals over time, before test (bt) and starting immediately after the FST. In the male control group, corticosterone levels rose immediately after the test (time = 0) and remained elevated for one hour (*F*(5, 48) = 30.50, *p* < 0.001). Two hours later, corticosterone levels decreased. Males exposed to Pb also exhibited increased corticosterone levels at 0 h (*p* = 0.033). However, the peak concentration occurred 0.5 h after the FST in this group (*p* < 0.001) and remained high for 2 h (*p* < 0.001). Furthermore, males exposed to Pb exhibited higher corticosterone levels than the control group (*F*(1, 48) = 39.17, *p* < 0.001; Fig. 6a).

In the female control group, corticosterone levels rose immediately after the test (time = 0) and remained elevated for one hour (*F*(5, 46) = 16.33, *p* < 0.001). However, levels decreased two hours later. Females exposed to Pb showed a peak concentration at 0 h (*p* < 0.001). Interestingly, corticosterone levels did not return to baseline values 24 h after the test (*p* = 0.04). Females exposed to Pb had higher corticosterone levels than the control group (F(1, 46) = 16.33, *p* < 0.001; Fig. 6b).

### Correlation Analysis

**Fig. 7:**
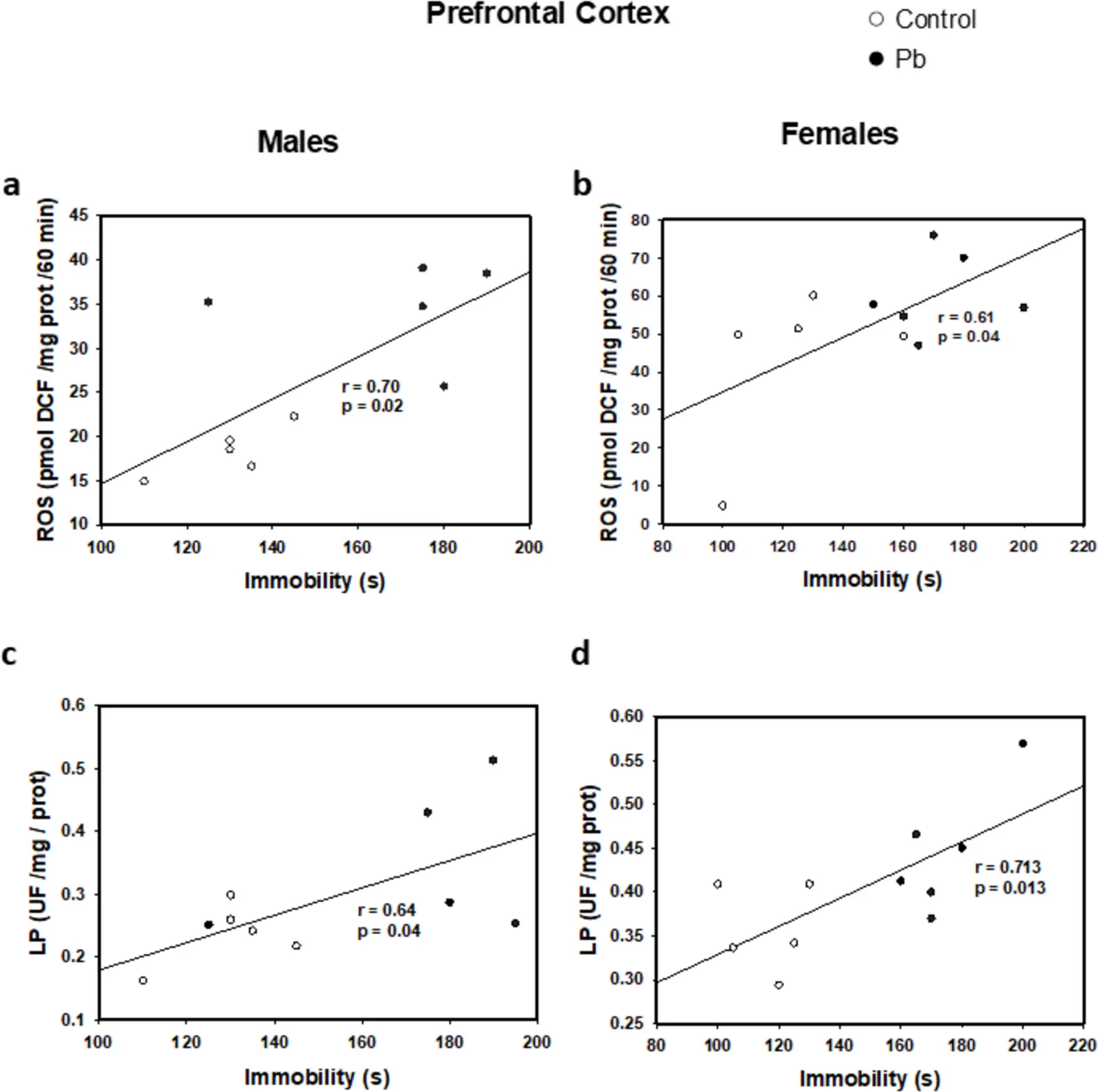
Correlation analysis between immobility time and oxidative damage levels in the prefrontal cortex. The correlation between immobility time during the forced swim test and ROS levels is shown in males (**a**) and females (**b**). The correlation of immobility time with LP levels is shown in males (**c**) and females (**d**). Open circles represent the control group, and closed circles represent the Pb-exposed group. Data were analyzed using a Pearson correlation test. *r*: Correlation coefficient

**Fig. 8:**
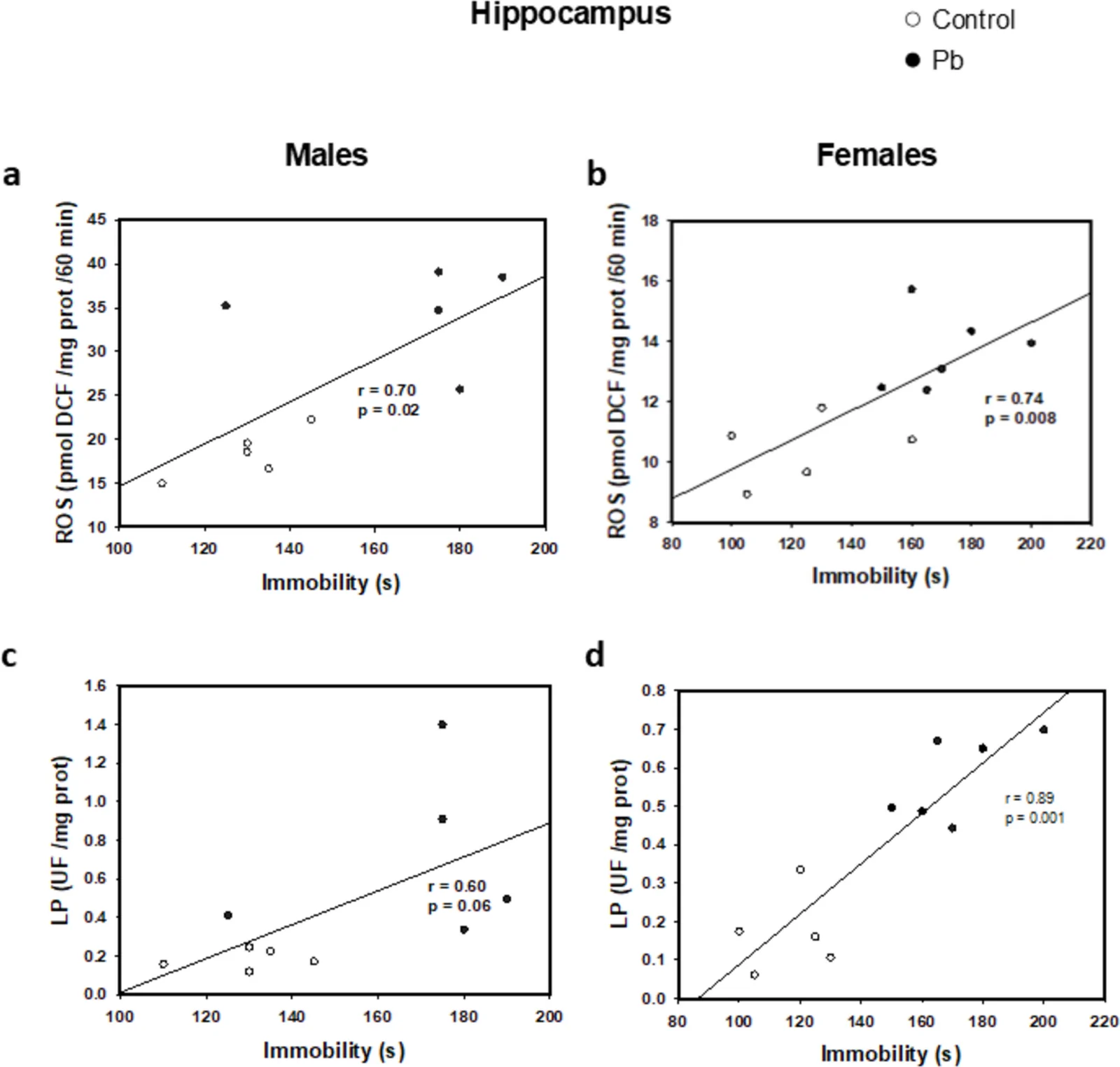
Correlation analysis between immobility time and oxidative damage levels in the hippocampus. The correlation between immobility time during the forced swim test and ROS levels is shown in males (**a**) and females (**b**). The correlation of immobility time with LP levels is shown in males (**c**) and females (**d**). Open circles represent the control group, and closed circles represent the Pb-exposed group. Data were analyzed using a Pearson correlation test

In males, positive correlations were found between immobility time in the FST and various parameters. These included ROS levels in the prefrontal cortex (*r* = 0.70, *p* = 0.02) and the hippocampus (*r* = 0.83, *p* = 0.002; Figs. 7a and 8a), as well as LP levels in the prefrontal cortex (*r* = 0.64, *p* = 0.04) and the hippocampus (*r* = 0.60, *p* = 0.06; Figs. 7c and 8c).

In females, positive correlations were found between immobility time and ROS levels in the prefrontal cortex (*r* = 0.81, *p* = 0.04) and the hippocampus (*r* = 0.74, *p* = 0.008; Figs. 7b and 8b), as well as between immobility time and LP levels in the prefrontal cortex (*r* = 0.713, *p* = 0.013) and the hippocampus (*r* = 0.89, *p* = 0.001; Figs. 7d and 8d).

## Discussion

The present study demonstrates that chronic Pb exposure from gestation through adulthood induces sex-dependent alterations in oxidative stress responses, corticosterone dynamics, and depressive-like behavior. By integrating behavioral, biochemical, and endocrine endpoints within a single experimental framework, the findings show the coexistence of oxidative imbalance and altered corticosterone responses following Pb exposure. Particularly, sex-specific differences in susceptibility were observed, with females exhibiting greater vulnerability to Pb-induced redox imbalance and a more prolonged corticosterone response following behavioral stress. These results highlight biological sex as an important factor influencing the neurobehavioral and neuroendocrine consequences associated with chronic developmental Pb exposure.

## Behavioral Alterations and Pb Accumulation

Our results confirm that chronic exposure to 250 ppm of Pb in drinking water is sufficient to induce significant accumulation of this metal in limbic brain regions. This aligns with the literature indicating that there is no safe threshold and that even lower doses (~ 50 ppm or less) trigger profound neurobiological alterations [22, 36]. In humans, greater loss of frontal cortex gray matter volume has been reported in males than in females following childhood exposure; experimentally, females have been shown to exhibit epigenetic changes (such as histone modifications) in the frontal cortex that are not observed in males [36]. Evidence indicates that Pb alters the trajectory of brain development differently in males and females via the glutamatergic system (NMDA and AMPA), selectively affecting the distribution of glutamate receptors in females. In the hippocampus of young females (PND 23), a significant increase in synaptic GluN2A and GluA1 subunits has been observed—an effect not seen in the same way in males [36]. Our results showed that the hippocampus exhibited substantial Pb accumulation in both sexes. However, female mice showed significantly higher Pb levels in the prefrontal cortex than males, suggesting regional and sex-dependent differences in Pb accumulation. Previous evidence supports biological sex as an important modifier of the neurobiological consequences of developmental Pb exposure. Tartaglione et al. [36] reported sex-dependent behavioral and molecular effects following developmental Pb exposure in rats, including differential alterations in hippocampal neuroplasticity-related endpoints. Similarly, Singh et al. [37] reviewed epidemiological and experimental evidence showing that developmental Pb exposure can produce sex-dependent effects on behavioral, molecular, genetic, and epigenetic outcomes. Importantly, these studies indicate that the direction and magnitude of sex differences are not consistent across experimental conditions and may depend on the developmental window of exposure, brain region, endpoint evaluated, and interactions among genetic, hormonal, epigenetic, and environmental factors. Thus, our finding of greater Pb accumulation in the female prefrontal cortex should be interpreted as a region- and sex-dependent effect under the present experimental conditions rather than as evidence of a generalized greater susceptibility of females to Pb neurotoxicity.

Behaviorally, exposure to Pb increased immobility times in both the TST and FST, suggesting a pro-depressant-like effect. Similar results have been reported in male rats [38, 39] and mice [39, 40]. Zhou et al. [41] included both sexes in their study; however, their results were not analyzed separately by sex. In the present study, the behavioral effect of Pb exposure was more pronounced in females, particularly in the FST. Previous studies have also demonstrated sex-dependent behavioral consequences of developmental Pb exposure [36, 37]. Tartaglione et al. [36] also showed that developmental Pb exposure produced behavioral alterations that differed between male and female rats, while subsequent work demonstrated that behavioral and hippocampal glutamatergic outcomes of developmental Pb exposure can also vary according to age and additional environmental factors [22]. Together, these findings support the interpretation that sex influences the behavioral consequences of developmental Pb exposure, but that the resulting pattern depends on the exposure conditions and biological endpoints examined.

According to Detke et al. [42], swimming behavior is indicative of serotonergic function, while climbing relates to catecholaminergic modulation. A selective decrease in swimming suggests that lead (Pb) interferes with serotonergic signaling. Although serotonin levels were not measured, future studies should quantify monoamines to confirm this hypothesis. It should be noted that motor tests revealed no differences between groups, confirming that behavioral changes were not confounded by motor deficits.

## Altered Corticosterone Responses Following Pb Exposure

Exposure to Pb altered the temporal profile of corticosterone following behavioral testing in both sexes. Altered glucocorticoid responses to stress have been associated with changes in HPA-axis activity and are frequently reported in affective disorders [43]. Haider et al. [43] reported that five weeks of exposure to 500 ppm Pb increased corticosterone levels in male rats. In contrast, Virgolini et al. [23] found that chronic exposure to moderate Pb concentrations (50 ppm) reduced basal plasma corticosterone levels in male rats. Taken together, these findings indicate that Pb exposure can modify corticosterone responses, with the direction and magnitude of these effects potentially depending on factors such as dose, exposure paradigm, and sex.

Sex differences were evident in the temporal profile of corticosterone release. Females exposed to Pb exhibited a rapid and prolonged corticosterone response that did not return to baseline levels within 24 h after the stressor, whereas Pb-exposed males showed a delayed but transient elevation that returned toward baseline within 24 h. These findings indicate sex-dependent alterations in the corticosterone response to behavioral stress following chronic Pb exposure, with females exhibiting a more prolonged response. Because corticosterone was the only endocrine endpoint evaluated, these findings should be interpreted as evidence of altered stress-related glucocorticoid responses rather than as a comprehensive demonstration of HPA-axis dysfunction. Future studies assessing additional components of the HPA axis, including ACTH and upstream regulatory mechanisms, would help characterize the neuroendocrine alterations associated with developmental Pb exposure.

## Oxidative Imbalance as a Central Mechanism of Pb Neurotoxicity

Oxidative stress plays a key role in the pathophysiology of depression [44, 45]. In this model, Pb exposure increased ROS and LP levels in brain regions related to mood, particularly the hippocampus. Even without behavioral testing, female animals showed elevated ROS levels in the prefrontal cortex, and both sexes showed increased LP levels in the hippocampus, revealing baseline oxidative vulnerability. After behavioral testing, ROS levels increased further in the prefrontal cortex, indicating that stress exacerbates Pb-induced oxidative damage to the cortex.

These findings contrast with reports of a stress-induced increase in oxidative stress in the hippocampus of male rats [46]. This indicates differences in redox regulation and regional antioxidant enzyme activity across species and sexes. In males, the combined effects of Pb exposure and behavioral stress increased cortical ROS and LP, which may reflect stronger antioxidant defenses given that males exhibit higher catalase activity in the prefrontal cortex [47]. Both sexes showed marked oxidative damage in the hippocampus, highlighting this region’s vulnerability to redox imbalance.

Correlation analyses reinforced these findings. In males, immobility time positively correlated with ROS and LP in the prefrontal cortex. In females, immobility time positively correlated with hippocampal ROS and LP. These sex-specific patterns of oxidative vulnerability suggest that, in males, cortical redox dysfunction is more relevant, while, in females, hippocampal oxidative stress predominates.

## Mechanistic Interpretation

Previous studies suggest that Pb exposure may affect the Nrf2/BDNF/serotonin pathway, potentially contributing to oxidative damage and behavioral alterations. Pb exposure has been reported to reduce Nrf2 signaling, which is a key regulator of antioxidant gene expression [48–50]. Reduced Nrf2 function has been associated with major depressive disorder and redox imbalance [51]. Conversely, antidepressants increase Nrf2 expression and antioxidant enzyme activity [52–55]. BDNF promotes serotonergic neurotransmission and mediates antidepressant-like effects [56–60]. Although these pathways were not evaluated in the present study, they provide a potential biological framework for future investigation of the molecular mechanisms associated with Pb-induced behavioral and redox alterations.

Aligned with the report by Muñoz-Castañeda et al. [61], we hypothesize that Pb-induced LP interferes with serotonergic transmission by compromising membrane integrity and receptor function [62]. Since ROS are short-lived, LP serves as a reliable indirect marker of oxidative stress. Pb increased both ROS and LP in the prefrontal cortex of both sexes (Figs. 4 and 5), which confirms previous reports of reduced antioxidant activity following Pb exposure [63, 64]. Females displayed higher basal ROS levels, indicating sex-related differences in redox status. To our knowledge, this is the first report quantifying both ROS and LP levels in the prefrontal cortex of Pb-exposed female mice. In the hippocampus, Pb increased ROS and LP levels in both sexes [63–65], confirming the region’s susceptibility to oxidative injury.

## Integrative Interpretation

Overall, females exhibited more pronounced depression-like behavior, accompanied by higher oxidative stress in selected brain regions and a more prolonged corticosterone response following behavioral stress. In contrast, males showed stronger associations between cortical oxidative markers and immobility, together with a less prolonged corticosterone response. These findings indicate that chronic developmental Pb exposure is associated with sex- and region-dependent oxidative alterations, changes in corticosterone dynamics, and depression-like behavior. Although oxidative markers were correlated with immobility, the present study did not directly assess the relationship between oxidative imbalance and corticosterone responses. Therefore, these processes should be considered concurrent features of Pb-induced neurotoxicity rather than mechanistically interconnected pathways based on the current data. Further studies are needed to determine whether and how redox imbalance and stress-related neuroendocrine alterations interact in the behavioral effects of developmental Pb exposure.

## Conclusions

Chronic Pb exposure from gestation to adulthood was associated with increased depression-like behavior, oxidative alterations in the prefrontal cortex and hippocampus, and changes in the corticosterone response to behavioral stress. These effects exhibited sex- and region-dependent patterns, with females showing greater vulnerability in several oxidative endpoints and a more prolonged corticosterone response following the forced swimming test. The associations between oxidative stress markers and immobility further support a relationship between redox imbalance and depression-like behavior. However, the present findings do not establish a causal or mechanistic relationship between oxidative alterations and corticosterone responses. Overall, these results highlight biological sex as an important factor influencing the behavioral, redox, and stress-related endocrine consequences of chronic developmental Pb exposure and support the importance of considering sex in neurotoxicological research. 

## Data Availability

No data sets were generated or analysed during the current study.
